# Healthcare providers’ knowledge, experience and challenges of reporting adverse events following immunisation: a qualitative study

**DOI:** 10.1186/1472-6963-13-313

**Published:** 2013-08-15

**Authors:** Adriana Parrella, Annette Braunack-Mayer, Michael Gold, Helen Marshall, Peter Baghurst

**Affiliations:** 1 Discipline of Paediatrics, School of Paediatrics and Reproductive Health, University of Adelaide, Adelaide, South Australia; 2 Discipline of Public Health, School of Population Health, University of Adelaide, Adelaide, South Australia; 3 School of Population Health, University of Adelaide, Adelaide, South Australia

**Keywords:** Adverse event following immunisation (AEFI), Surveillance, Healthcare provider, Reporting, Qualitative

## Abstract

**Background:**

Healthcare provider spontaneous reporting of suspected adverse events following immunisation (AEFI) is central to monitoring post-licensure vaccine safety, but little is known about how healthcare professionals recognise and report to surveillance systems. The aim of this study was explore the knowledge, experience and attitudes of medical and nursing professionals towards detecting and reporting AEFI.

**Methods:**

We conducted a qualitative study, using **s**emi-structured, face to face interviews with 13 Paediatric Emergency Department consultants from a tertiary paediatric hospital, 10 General Practitioners, 2 local council immunisation and 4 General Practice nurses, recruited using purposive sampling in Adelaide, South Australia, between December 2010 and September 2011. We identified emergent themes related to previous experience of an AEFI in practice, awareness and experience of AEFI reporting, factors that would facilitate or impede reporting and previous training in vaccine safety. Thematic analysis was used to analyse the data.

**Results:**

AEFI reporting was infrequent across all groups, despite most participants having reviewed an AEFI. We found confusion about how to report an AEFI and variability, according to the provider group, as to the type of events that would constitute a reportable AEFI. Participants’ interpretation of a “serious” or “unexpected” AEFI varied across the three groups. Common barriers to reporting included time constraints and unsatisfactory reporting processes. Nurses were more likely to have received formal training in vaccine safety and reporting than medical practitioners.

**Conclusions:**

This study provides an overview of experience and beliefs of three healthcare professional groups in relation to identifying and reporting AEFI. The qualitative assessment reveals differences in experience and awareness of AEFI reporting across the three professional groups. Most participants appreciated the importance of their role in AEFI surveillance and monitoring the ongoing safety of vaccines. Future initiatives to improve education, such as increased training to health care providers, particularly, medical professionals, are required and should be included in both undergraduate curricula and ongoing, professional development.

## Background

In Australia, the spontaneous reporting of adverse events following immunisation (AEFI) is the primary mechanism used for post-marketing passive surveillance (PMS) of licensed vaccines. Passive AEFI surveillance is common in many countries, worldwide [1-4]. The process relies on immunisation providers, health professionals, and consumers voluntarily submitting ad-hoc reports to jurisdictional public health and/or federal regulatory authorities [5]. Vaccine manufactures are mandated to report to the federal authority, the Therapeutic Goods Administration (TGA), and in four of the eight Australian states/territories, but not South Australia, health professionals are mandated by jurisdictional legislation to report to the local public health authority. At the federal level up until 2013, the Advisory Committee for Safety of Medicines (ACSOM), a subcommittee of the TGA was responsible for the ongoing evaluation of all drug and vaccine safety. As of 2013, in response to recommendations for an improved system of governance for vaccine safety monitoring [6], a new statutory Advisory Committee on the Safety of Vaccines (ACSOV) has been established to evaluate vaccine safety. Any medical events occurring after vaccination, that are regarded as “serious” and/or “unexpected” should be reported [7,8]. An established causal association with vaccination is not a pre-requisite for reporting [9].

Effective PMS is critical for a number of reasons. First, for new vaccines, pre-licensure clinical trials are not powered to detect rare adverse events that occur with a frequency of less than 1 in 1,000, or with delayed onset, and they are usually tested in homogeneous, healthy study populations [3,10]. Thus, PMS aims to identify potential safety signals which may require further investigation not identified in pre-licensure trials and that may become apparent outside the controlled conditions of clinical trials. Secondly, for established vaccines, PMS aims to monitor known adverse reactions and if the observed rate exceeds the expected rate, further investigation is required. Finally, PMS should detect program errors, such as incorrect vaccine administration or manufacture [11,12]. Hence, all licensed vaccines require specific pharmacovigilance plans that incorporate post-licensure passive surveillance and are “timely, efficient, sufficiently large and in place for the life of the vaccine” [11]. An example of the importance of voluntary reporting of suspected AEFI was demonstrated by the withdrawal of the Rotashield vaccine in the United States in 1999. Ten months post-licensure and following 1.5 million doses administered, 15 reported cases of intussusception, higher than expected to occur, signalled the need for suspension of its use and further evaluation of the vaccine [13].

Under-reporting is a known limitation of passive vaccine and adverse drug reaction (ADR) surveillance systems [12,14]. In Australia this is demonstrated by the marked variation of AEFI reporting rates across jurisdictions for the same vaccines [15,16]. The importance of and need for timely healthcare provider reporting of AEFI as they occur was highlighted by a recent Australian experience of a vaccine safety signal. On the 23rd April 2010, a seasonal trivalent influenza vaccine (STIV) for children aged less than 5 years was suspended nationally for three months due to an increased incidence of fever and febrile convulsions, [17] associated with the vaccine brand, Fluvax (CSL). In an analysis of AEFI reports submitted to the South Australian Department of Health in the first six months in 2010, the majority (71%) of influenza AEFI reports submitted by healthcare providers were received *after* the STIV program was suspended [18]. Subsequent reviews of AEFI surveillance in Australia following the STIV suspension have suggested that under-reporting and delayed reporting of febrile convulsions, contributed to delays in signal detection [6,19].

Healthcare professional AEFI reporting is an under-researched area, with only four studies conducted elsewhere, published to date [20-22]. All four studies employed quantitative methods to either measure awareness of surveillance, reasons for reporting or to compare actual AEFI reports by health professionals. The first examined Canadian family physicians’ awareness of vaccine safety monitoring systems and reporting frequency for vaccine associated adverse events [20]. Less than half of the study respondents were aware of a monitoring system for AEFI, only one third knew of reporting criteria and only one in four had received vaccine adverse event education during medical training. The primary reason for not reporting was that an AEFI was never observed, the respondents did not know reporting was expected, the event did not seem serious enough or respondents were not aware of reporting procedures. Ranganathan et al. (2003) examined AEFI reports of Meningococcal serogroup C Conjugate (Men C) vaccine submitted to the Yellow Card Scheme (United Kingdom) by hospital doctors, General Practitioners (GPs) and nurses [22]. This study found nurses reported AEFI more frequently compared with GPs and hospital doctors and that completeness of the reports varied across the professional group. The third study of health professional AEFI reporting conducted in the United States included physicians, pharmacists, and nurses [21] and examined the frequency of reporting to the Vaccine Adverse Event Reporting System (VAERS), beliefs and awareness of AEFI reporting, barriers to reporting and strategies to increase reporting rates. Of all respondents, 71% had never reported an AEFI, with 17% indicating they were not aware of how to report. The study demonstrated significant differences in having ever reported an AEFI by health professional type. Barriers to reporting included unclear definitions of a reportable AEFI, time pressures in completing a report, and confusion in whose responsibility it was to report. Reporting was associated with being alerted to look for specific events, discounting other explanations for the adverse event; observing the same AEFI repeatedly and whether the events occurred in vulnerable patient groups such as pregnant women, infants or patients aged ≥65 years. The fourth study is the most recent conducted to date and included family physicians, physician assistants, nurse practitioners, practice nurses and nurses working in paediatrics, family medicine and internal medicine [23]. The survey assessed demographics and professional characteristics and knowledge and attitudes toward identifying and reporting an AEFI to the Vaccine Adverse Event Reporting System (VAERS) in the United States. Although nearly three quarters of study participants were familiar with VAERS, only 14% were “very” or “extremely” familiar with the paper reporting procedure and approximately one third were not familiar when it was required to report an AEFI. Approximately 40% of all study participants had identified at least one AEFI, with only 18% indicating they had reported to VAERS. Respondents indicated they would report serious AEFI regardless of whether they were known (73%) or unknown (62%) to be associated with immunisation. Those who indicated that they were not familiar with submitting a paper report to VAERS were more likely not to report than those who were familiar with the process. Similarly, respondents who were not at all familiar with reporting criteria to VAERS tended not to report compared with those who were familiar with the requirements.

Studies of adverse drug reaction (ADR) reporting by health professionals have identified several factors that are common to under-reporting of AEFI. Ignorance of reportable events, lack of awareness of a reporting system, insecurity regarding causation (not possible to ascertain whether the drug caused the reaction) and lack of time are common reasons associated with lack of reporting [14,24-28]. Other factors not demonstrated in previous AEFI studies that have been associated with under-reporting of ADR include fear of litigation; indifference; lack of financial incentives to report and a belief that only safe drugs are released into the market [24,25]. Some parallels exist in adverse medical incident reporting studies. These studies reveal parallel differences in reporting behaviour between medical specialties where nurses are more likely to report to internal incident reporting systems than doctors and ‘the fear of blame’ as a common barrier to reporting by doctors [29-31].

Consumer perceptions and experience of health professional ADR reporting have demonstrated concern that health professionals’ lack of clarity in recognising adverse medicine events prevents reporting of potential adverse events [32,33]. Two recommendations arising from a meeting of the Consumers Health Forum of Australia (CHF) in June 2011 were to improve and encourage adverse event reporting processes through training and education for health professionals [34]. These recommendations are echoed in a national review of Australian AEFI surveillance following the 2010 STIV safety signal to increase both consumer and health professional awareness of AEFI reporting and to improve communication and notification of AEFI between jurisdictional and federal health authorities [6].

It is likely that differences in healthcare provider AEFI knowledge and practice of reporting results in inconsistent adverse event data collection and, ultimately, inaccurate measurement of the incidence of vaccine adverse events, by delaying or missing important vaccine safety concerns [35]. Since spontaneous reporting is central to passive vaccine PMS and given that health professionals provide the majority of AEFI reports to surveillance systems [36], it is important to understand not only the factors such as awareness of and frequency of reporting, but also how health professionals identify/conceptualise a reportable AEFI. This paper presents results of a qualitative study that aimed to determine how healthcare providers identify and report AEFI within the South Australian context.

## Methods

### Design

Following a review of key findings from existing literature on AEFI and ADR reporting as described above and information obtained from a study of parent AEFI reporters we had previously conducted [37], we chose to adopt in-depth qualitative interviews for the study design as it was most suited to our research questions: What are the experiences, awareness and knowledge of healthcare providers in AEFI reporting and how do healthcare providers conceptualise a reportable AEFI? These questions and the associated study design are consistent with a social constructionist paradigm in qualitative research, enabling the interviewer to make meaning of each participant’s “world”, their individual perspectives and meanings in a context that is shaped by their organisational environment and broader social structures [38].

We chose to conduct individual, face to face interviews as appropriate to examine each participant’s specific experience and understandings of AEFI and because it was most suited to participants’ work schedules. The interviews were conducted with the General Practioners (GPs) and Paediatric Emergency Department (PED) consultants, between December 2010 and February 2011. Based on preliminary analysis of the interview data, it was recognised that nurses also played an important role in AEFI reporting, and a further six interviews were conducted with two local council immunisation and four general practice nurses in September 2011.

### Recruitment

We recruited twenty-nine healthcare professionals from an Emergency Department of a tertiary, paediatric hospital, GP clinics, and local council immunisation clinics in Adelaide, capital city of South Australia (population 1.6 million). Characteristics of participants are presented in Table 1. Purposive sampling was used to identify participants for the study [39]. The participants recruited in each category (see Table 1) represented a range of health professionals who were in a position to detect, manage and/or report an AEFI. The PED consultants were recruited via the Emergency Department with initial information about the study communicated to participants via the head of Emergency. All consultants except one agreed to participate. We used three strategies to recruit the GPs including contacting potential participants via professional (university research academics and clinical) contacts of the authors, advertising via an electronic distribution mail list of the local branch of the Royal Australian College of General Practitioners and finally via electronic communication within an academic organisation involved in training general practitioners. The study nurses were recruited via contacts of the authors, the general practice clinics involved in the study, and via the local Department of Health Immunisation Section.

**Table 1:** Study participants

**Professional group**	**Female**	**Male**	**Age range (years)**	**Mean number of years worked in professional group**
Nurse	6	0	31-53	19
Paediatric Emergency Department specialist	6	7	35-57	15
General Practitioner	8	2	40-57	21

### Topic Guide

The semi-structured, open-ended interviews were conducted using a topic guide (see Table 2). The original interview schedule was developed from a review of key findings of literature surrounding AEFI and ADR reporting as described earlier. Each interview sought to explore participants’ knowledge and experience of detecting, managing and reporting an AEFI; factors that would facilitate or impede AEFI reporting; understanding of AEFI surveillance and previous training in vaccine safety. All interviews were conducted at participants’ workplace, ranging from 25 to 65 minutes.

**Table 2:** Interview topic guide

**Theme**	**Guiding question**
**Experience of an AEFI**	1. Could you tell me about an AEFI you have seen during the course of your work?
	2. How often have you seen an AEFI in this workplace or during your career?
	3. How did you respond to the AEFI?
	4. How did the event turn out?
**Reporting an AEFI**	1. Have you ever reported an AEFI? Why?
	2. How have you reported?
	3. Was it an easy/difficult process? Could you explain?
	4. If you talked to an authority about the event what was the response from the person?
	5. If you needed to report an AEFI today how would you do it?
	6. What do you think are the main factors that would lead you to report an AEFI?
	7. Why would you report an AEFI?
	8. What would you not report as an AEFI?
	9. What would be your preferred format for reporting? Why?
**Workplace**	1. Can you tell me about whether AEFI are discussed with your colleagues?
	2. Could you describe any policy/protocol for reporting an AEFI in your workplace?
**Surveillance**	1. Could you describe your understanding of how vaccines are monitored for safety after they are released to the public?
	2. Who do you think should be responsible for monitoring vaccine safety in Australia?
	3. How do you access communication regarding vaccine safety issues?
	4. Is there sufficient information available to you from surveillance authorities or other sources? Explain.
	5. In your opinion, who should be responsible for monitoring the ongoing safety of vaccines?
	6. What do you think happens after an AEFI report is made?
	7. What is your impression of how safety is monitored?
**Training**	1. Could you tell me about any training you have had in vaccine safety either during your career or as a student?
	2. How do you update your knowledge in vaccine safety?
	3. What would be an ideal way to update or provide training?
	4. Do you think doctors and nurses have sufficient training and knowledge in current vaccine safety issues? Why?

### Ethical considerations

Participation was voluntary and signed, informed consent was obtained before conducting the interviews. The study was approved by the University of Adelaide and Children Youth and Women’s Health Service (CYWHS) Human Research Ethics Committees. This study adhered to the qualitative research review guidelines (RATS) [40].

### Analysis

Each interview was audio-taped and data transcribed verbatim by AP. Thematic analysis was used to structure analysis of the transcripts [41] with NVivo, version 9 (QSR International, UK). Initially, open coding of interview data was undertaken. These codes were generated inductively from participants’ descriptions of their experiences in responding to and reporting an AEFI, and awareness of vaccine safety surveillance. Following initial coding of transcripts, preliminary themes that captured information relevant to the research questions were generated. This process involved identifying patterns in the data: recurring ideas, perspectives and descriptions that depicted each participant’s context and perspective. The final analysis for this study focussed on key themes, narratives, and professional histories emerging from the interviews. Data concordance was verified by AP and ABM, a trained qualitative researcher with extensive experience in medical and public health qualitative research. Key themes were discussed with the research team that included two clinicians with expertise in vaccine safety and surveillance (HM and MG) at regular team meetings. We achieved topical saturation as similar themes emerged from various participants from each professional group after preliminary analysis of initial interviews. Quotes that best illustrate important representation of participants’ views and experiences identified through our iterative process of review and discussion are presented in the following section.

## Results

### Previous experience of an AEFI and reporting

Most participants (27/29) reported seeing or being involved in the care of children or adults with a suspected AEFI, in their current or previous workplace. The cases included children presenting with suspected hypotonic hypo-responsive events, anaphylaxis, febrile convulsion, non-febrile convulsions, extensive limb swelling, high fevers and skin rashes (reported as allergic events). Although participants described experience of at least one AEFI throughout their career, most stated they were “rare” or “not that common”, and occurred “years” prior to the interview. The most recent events recalled were febrile convulsions following STIV vaccination in 2010 and a “severe local swelling” in the week prior to the study interview.

*“I haven’t had a lot of adverse reactions at all. They’re quite rare actually. If you think of the number of kids we vaccinate. I’ve had lots of local reactions but I don’t recall off the top of my head any significant.”* GP 9

*“We’d still be seeing the reactions rather than but no, not very common at all. Even less since we’ve used the acellular vaccine, even less.”* GP 7

Of all participants, 19/29, (7 GPs, 5 nurses and 5 PED consultants), indicated they had reported an AEFI to a surveillance system at some point in their career, either in Australia or overseas. Only two participants stated they had reported more than once, despite the fact that most had worked for many years in the health system (a mean of 18 years for all three groups). When asked to recall when they had reported, a common response was in the distant past, with some as far back as “fifteen or twenty years ago.”

*“That one with the measles I would have reported. I think there was a couple of others too but it’s going back a long way.”* GP 7

### Awareness of reporting

All nurses were familiar with paper and telephone reporting procedures to the local Department of Health and also described their workplace reporting processes, such as having the report forms on hand and/or an existing protocol for reporting adverse events (Table 3). Six of the thirteen PED consultants (46%) stated they were not aware of a system for reporting or how to report an AEFI.

*“I would probably have to ask my colleagues how to do it.”* ED 4

*“I’d have to ask one of the other consultants what the procedure was, because I don’t currently know.”* ED 9

**Table 3:** Participants’ awareness of AEFI reporting protocol or policy in their work setting

**Nurses**	**General practitioners**	**ED consultants**
If we see an adverse event, then we do report. We have the forms for reporting. (General Practice nurse)	I think I would say that you know the majority that would be 99%, is done by our nurse and would probably only get reported from the nurse	I’d have to double-check. I’d have to ask a colleague
I would say there’s one in the policy manual. (General Practice nurse)	No I don’t know that there is one here actually.	We can just click on forms, adverse events reporting form and just print it out, so that’s what we do.
The forms are in our filing cabinet. But I know you can get it from SAICU and I know it’s on their website. We’re actually in the process of doing a procedure, protocol. (General Practice nurse)	I would say there wouldn’t be anything completely formal that we’ve ever discussed at a meeting or anything. I don’t think there’s ever been a formal policy. No.	*Not answered*
Not actually in writing but because I’m the only one here generally, anything that’s out of the normal goes past me anyway. We always keep a copy of them (the adverse event reporting form) at the clinics. (Council immunisation nurse)	Not that I’m aware of. There may be, but not sure.	I don’t really know because I’ve never had to do it because obviously it’s quite rare.
I do the reporting and advise the doctors that I’ve done that as well. If we do any written documentation it’s always scanned into the notes too. (General Practice nurse)	No actually we don’t as far as I know have a policy. Probably we should, but no we don’t.	I would have to look at information on our intranet that has information about reporting adverse reactions to vaccines and remind myself how to do it.
It is in our standard operation procedure that we do have that, if an adverse event occurred, it just says fill in a form. (Council immunisation nurse).	Well our practice nurse looks after all these things and she would report.	I remember looking up a number probably from the Immunisation Handbook.
	No not specifically. There hasn’t been a designated discussion about what we do about these things when they occur.	I don’t think so. I’m not familiar with a documented protocol as such.
	There’s those blue forms.	I’d have to ask one of the other consultants what the procedure was, because I don’t currently know.
	Not formal, but we know to report to ADRAC.	We’ve got it on our web on our intranet there’s links to it. The numbers there or you make the notification or you just fill it in and send it off.
	I’d have to see what the protocol was, but we haven’t had one for so long	Reporting would not be protocolised.
		We’ve got the blue forms. We fill in the blue forms and send them off.
		No, there is no protocol

Two GPs were not aware of how to make a report, even though one stated having reported previously. The second GP had previously diagnosed an AEFI which would have been reported, had she known of a reporting system.

*“I found it difficult to try and find out where I was meant to report and then due to competing demands didn’t seek further information.”* GP 5

For those who indicated awareness, reporting was thought to occur generally either via the national adverse drug reporting system or the local Department of Health. Few participants indicated awareness of both national and local reporting systems. We found participants were generally confused about the various reporting options and unaware that reports could be notified via phone, postal, fax, electronic or online submission.

*“It would be helpful if the practice nurses could report on my behalf.”* GP 10

*“It would be nice to have a number, a telephone number with who to go to. That’s the sort of thing we probably need with adverse events to vaccines.”* GP 6

When describing awareness of workplace policies, participants were also prompted to describe whether AEFIs were discussed during the course of their work. If an AEFI was discussed in the various workplace settings, it would usually occur informally with colleagues if a patient presented with symptoms that were unusual or serious. For example, in the hospital setting, around the time of the influenza safety signal in 2010, the ED consultants recalled informal discussions with colleagues of febrile convulsion cases presenting to the ED. The nurses would discuss cases that were “out of the norm”.

“We do discuss it between us quite a lot if you get something quite a bit different. You know such and such happened have you had that happen with yours or are you aware of that being anything? So we do usually discuss it amongst ourselves.” GP 6

“We tend to talk about things that happen. If it was something serious I think generally we would discuss those things.” GP 7

### Recognition of a reportable AEFI

Participants were asked to describe the types of events they would consider necessary to report. All stated that a reportable AEFI was an event characterised as “serious” and/or “unexpected”. Reactions were generally considered serious if they were life-threatening (such as anaphylaxis); clinically significant or severe (for example, convulsions); and/or relevant to the patient’s future vaccinations, because of the potential impact on future vaccination decisions.

*“I’ve never seen an anaphylaxis. I’ve never seen a hypotonic reaction. I’ve never seen anything I would classify as serious. Ever. I’ve never seen an AEFI that I’ve had to report.”* GP 3

*“Most of the cases that present actually aren’t significant events. So, that would be the usual fevers following vaccinations or localised reactions. Few actually meeting the criteria for being significant. I’ve not seen anyone with an anaphylaxis.”* ED 9

Two underlying interpretations were evident when participants described an “unexpected” AEFI. In the first instance, “unexpected” referred to an event that was rare, but with a known (but low) probability of occurring, such as anaphylaxis. These were regarded as unexpected because they were more severe and less common than the “normal” vaccine reactions.

*“Beyond the reasonable in terms of you know what you would expect. It’s obviously more severe.”* Nurse 3

When compared by professional group, all GPs and nurses would report this type of unexpected AEFI, (severe or rare, but previously recognised), whereas only half of the PED consultants explicitly stated or implied this. Discussion regarding febrile convulsions illustrated a difference in interpretation of “unexpected” across the groups. Most PED consultants stated that they had managed children who had experienced febrile convulsions in relation to influenza vaccination in 2010; however, only three could recall reporting this as an AEFI. When discussing the 2010 safety signal and opinions of why febrile convulsions were not reported, several reasoned that they are a known AEFI, that the children had experienced relatively minor convulsions, and that only prolonged convulsions that were “clinically significant” should be reported.

*“I guess you know we saw a number of children not long after the vaccine was released with febrile, apparent febrile reactions to the vaccine who didn’t appear to be particularly otherwise unwell. I guess febrile reactions to vaccines are relatively common, that we weren’t particularly perturbed about it at all until there were reports of children becoming quite unwell and having prolonged convulsions and there is significant morbidity associated with those, particularly interstate. I’m not sure whether there were terribly many in Adelaide.”* ED5

The PED consultants tended to describe as reportable only those events that were very severe or life-threatening, often referred to as “clinically significant” or “dangerous.”

*“I think it has to be a very significant event… where it’s well above the normal thing and potentially quite dangerous.”* ED 6

The second meaning attributed to “unexpected,” was a reaction that was not known to occur following vaccination. This type of AEFI would be reported because there was no established scientific evidence available that connected it to a vaccination.

*“If a child came back the next day or a week later and had an illness or an event that I couldn’t in my mind relate necessarily to the vaccine then yes I would.”* GP3

In addition to serious and unexpected reactions, some participants considered all adverse events occurring following newly released vaccines should be reported. Three participants stated all reactions, regardless of severity, should be reported.

*“Well I guess theoretically any reaction to a vaccine should be notified, even if it’s a minor reaction. The flu vaccine was a good case in that although we saw the children as having relatively minor febrile reactions to the vaccine there was obviously children who were having more severe end of the spectrum reactions associated with fever, so it’s a good illustration that probably any reaction to a vaccine should probably be notified.”* ED 1

*“I think any adverse event no matter how little or large needs to be reported.”* Nurse 2

### Barriers to reporting

When discussing vaccine safety surveillance, most participants stated the critical role of healthcare providers in reporting AEFI but also recognised the limitation of passive surveillance of relying on healthcare providers to report.

*“If you don’t have reports you don’t know how it’s going out there in the arena, do you?”* Nurse 6

*“Well I think everyone involved in administering vaccines which includes GPs and nurses and anybody seeing people. So, it’s really, all of us have to play a role.”* GP 4

*“It seems to be more clinical adverse reactions are heavily dependent on the clinician reporting them.”* ED 10

*“There’s a lot of assumptions made. We’re assuming someone’s going to tell us and then we’re assuming that we’re going to notify someone else when we find out.”* GP 9

Although reporting by health professionals and the public was understood as key to monitoring AEFI, two participants did not believe they shared responsibility for AEFI surveillance.

“Not me. I don’t know. There’s probably some immunisation body.

*The only way you’re going to know that there’s some problem with a vaccine is if people are going to report significant events post –vaccine. And then someone else can sort it out.”* ED3

*“I think it should be a government department because it’s a public health issue.”* GP5

The amount of information required in completing a report, time constraints, competing workplace priorities in the workplace and dissatisfaction with reporting methods were identified as barriers to reporting by the GPs and PED consultants. By contrast, the nurses did not describe any of these barriers.

*“Busy clinicians really don’t have time to sit down and fill out several pages of report form.”* ED 1

*“The reporting system is too difficult. The only way I want to report anything is automatically through my software.”* GP4

### Preferred format for reporting

Participants’ preferences for a preferred format for reporting an AEFI varied across the three professional groups, and covered the current options for paper, phone, fax and electronic reporting. The nurses preferred either the phone or paper reporting, the GPs, phone or web-based reporting and the PED consultants varied in their opinions, stating paper/fax, phone and electronic formats. The phone was often stated as a convenient method for communicating and receiving immediate feedback/response with an immunisation professional. Paper reporting was believed useful to have a record or “trail” of communication with the local Department of Health and for the purpose of having patients’ events recorded with their medical history.

*“I prefer phone because I can ask questions for myself, as for if there was any correlation. So I’m reporting the incident but also following-up the information for myself or for the patient.”* Nurse 3

When discussing ideal electronic formats, GPs and PED consultants suggested creating systems that were linked to their workplace systems/practice management software and allowed for automatic submission.

*“With electronic, particularly if there is a system built into your database using emergency as an example, if there is a reporting form built into the system so that it’s prepopulated with demographic data and all you need to do is click some boxes, the form would be sent in automatically.”* ED 5

### Training

All nurses had received some formal training in vaccine safety and AEFI reporting, such as Division of General Practice, Department of Health workshops, or post-graduate university training for immunisation providers. Most of the GPs and PED consultants could not recall specific training either pre- or post-graduation. All GPs and most PED consultants believed that, in general, doctors’ pre-service education in vaccine safety and adverse event reporting was inadequate.

*“I can’t remember having any specific training on immunisations or reporting and adverse reactions. It’s just assumed that we have obtained that knowledge somewhere rather than actually having a specific study or certification in vaccine.”* ED 8

*“I would have to say I don’t think I had any training as a student at all. Or at least can’t recall it. I can’t remember hearing anything about vaccinations in medical school apart from sick people with COPD need vaccinations but nothing about the vaccines or safety.”* GP 5

All participants supported strategies for updating knowledge via the continuing medical education programs of their relevant professional accreditation organisations.

## Discussion

This is the first qualitative study, to our knowledge, that explores healthcare provider AEFI reporting awareness, practices and attitudes. We found reporting was infrequent across the three groups interviewed and conflicting views between groups as to what events would constitute an AEFI. Potential reasons for this could be that an AEFI occurs infrequently; that an AEFI is not recognised as such; and/or that an AEFI is recognised, but not reported. Our results show events which were either completely unexpected (that are not known to occur following vaccination), or which might represent an increase in expected reactions were less likely to be reported.

This study has shown that the requirement for all “serious” events to be reported to authorities, regardless of whether they were causally related to the vaccination, was interpreted differently amongst participants and by professional group. All participants would report the most severe events, often termed “life-threatening,” or “dangerous.” However, we found from the PED consultant interviews that, on the whole, they would only report events that were perceived as “life threatening.” Compared with the nurses and GPs, they were less likely to report other events that were not as severe and those that are a known AEFI. The under-reporting of febrile convulsions following STIV in April 2010, could possibly be an illustrative example. Based on these interviews, we could reason that the PED consultants did not report febrile convulsions because this is a known complication of immunisation associated with fever. Taken together with the belief that most of the children they treated had experienced minor, (or not clinically significant) convulsions illustrates their differing interpretation of “serious” compared with GPs and nurses. Possibly, working in an environment in which one regularly sees serious and life-threatening presentations, compared with other settings, such as an immunisation clinic or family physician’s workplace, increases a hospital emergency doctor’s threshold for the definition of “severe” or “serious” and, therefore, what would be reported. Viewed in this light, under-reporting can be explained partly by the varied interpretation of what constitutes an AEFI.

The context of the workplace setting in this study is important to consider in relation to understanding factors that might influence a health professional’s decision to report an AEFI. We did not seek information from each work setting involved about whether in fact there was an established policy or protocol for reporting. However, from the interviews we conducted, it was apparent that reporting was an established norm for immunisation nurses in local council clinics, as a council nurse’s core work is providing immunisations to the public. Having report forms at hand and documented protocols for AEFI reporting facilitated reporting in such settings. We suggest there are three possible explanations for the variations in awareness of participants from the general practice and hospital settings (Table 3). First, it may be that there was no current policy in place. Second, if a policy existed, it had not been introduced or established effectively within the workplace. For example, in the hospital setting, the ED consultants did have access to the local Department of Health reporting form via the internal intranet; however, few indicated awareness of it during interviews. This would suggest a need to ensure staff are informed and updated about accessing the reporting link. Given that the study occurred less than 12 months after the safety signal associated with the seasonal influenza vaccine and subsequent relay of public health alerts to hospitals and primary healthcare settings regarding the occurrence of febrile convulsions and need to report, it was surprising that there were such low levels of awareness. A third explanation for low levels of awareness amongst the GPs and ED consultants could be that reporting was not seen as a prime function of medical staff and might be delegated to nursing or administrative staff. Apart from one GP who indicated that the nurse at his practice would be responsible for reporting as part of her role in immunising patients, we found no evidence of delegated reporting amongst the GPs. In the ED setting, delegating the reporting to a registrar who was undertaking an ED rotation was described by some consultants and hence could explain their unfamiliarity with the actual processes of reporting, regardless of whether it was to local or national surveillance authorities. In this context one could speculate that reporting was not seen as a primary function of the clinician, but rather an administrative function to be performed by non-medical staff or, as in the ED setting, junior medical staff.

Despite the limitations of passive surveillance, it is not likely to be replaced by alternate methods of surveillance that do not rely on healthcare professionals’ awareness or readiness to report, such as data linkage [42-44]. Passive surveillance should monitor vaccine safety and detect safety signals in real time or near-real time. Alternate methods of surveillance such as data linkage or sentinel surveillance are usually used to detect known events and to test hypotheses for associations between a vaccine and an AEFI [45] but are limited by timeliness of reporting. Thus, there is an ongoing need for robust passive AEFI reporting systems. From this study it is clear that, even if an AEFI is recognised, there are significant barriers to reporting by health care providers. These barriers are consistent with factors identified in previous studies of AEFI and ADR reporting [20,21,24] and include a lack of awareness or confusion about reporting systems, a lack of time to report and differing perceptions of a reportable AEFI. Unlike other studies, we did not find evidence about fear of litigation, or that vaccine adverse events are not reported because of inherent trust that licensed vaccines are all safe [24].

Few participants in this study were aware of both local and national reporting processes. Future research should explore whether a single pathway for AEFI reporting may be preferred by healthcare providers, rather than the existing system which provides a choice between reporting to the local Department of Health within each Australian jurisdiction or the national body (TGA). We also found differing preferences for the varied methods of reporting. In addition to these barriers, under-reporting may in part be attributed to the administration of less reactogenic vaccines in more recent years that has resulted in lower occurrence of some reactions. This would result in less awareness of reporting, as practitioners are less likely to be familiar with a system if they do not need to use it. However, this would not explain the differences in awareness across the three professional groups, as nurses were more familiar with both reporting processes and specific workplace protocols for reporting.

We found the nurses were more likely than the doctors to have received formal training in vaccine safety surveillance, which generally occurred as professional development training. This finding is consistent with previous published studies that reported low levels of vaccine safety education during medical training in Europe and Canada [20,46] and an unpublished, cross-sectional survey of 452 GPs, GP nurses, midwives, paediatric and community nurses, conducted in Sydney, Australia [47]. Our study confirms a need to provide adequate education across healthcare providers’ training, both pre- and in-service, which has been recognised internationally [46,48]. Adverse event reporting should be incorporated in continuing medical education programs.

The strength of this study lies in its qualitative approach. This format allowed participants to provide detailed accounts of their experiences and understanding of AEFI and reporting system. We were also able to sample participants from different work settings and professions. However, there were some limitations that may affect the generalisability of our findings beyond this study. Firstly, our participants all came from the one jurisdiction, and their responses may have been shaped by the organisational context for reporting of AEFI in that jurisdiction. Second, we acknowledge that participants in this study self-selected to participate and may provide an element of responder bias, since more motivated individuals or those with an interest in immunisation may have participated. However, we expected that responder bias would have been associated with greater familiarity with AEFIs and reporting systems than was evidenced in our study. Third, most participants had completed undergraduate training decades previously and this may have influenced their recall of adverse event training.

Our findings support the recommendations of the two reviews into AEFI surveillance in Australia, which were initiated because of the occurrence and delayed detection of febrile convulsions post STIV in 2010. Both reviews highlighted deficiencies in healthcare provider reporting [6,19] and recommend a need to improve AEFI detection and reporting by introducing strategies aimed at increasing awareness of national reporting and strengthening communication within the surveillance system. Future research that would inform strategies to improve AEFI reporting should aim to include the perspectives of workplace managers and surveillance authorities as key informants.

## Conclusion

Although the majority of participants had observed an AEFI in clinical practice and understood the importance of their role in AEFI reporting for post-marketing safety surveillance, we found reporting was infrequent. Reporting was related to the perceived interpretation of a reportable AEFI. The current guideline of reporting “serious” and “unexpected” events was interpreted differently in the three work settings and this would suggest there needs to be clearer definition and guidelines about reportable adverse events. Barriers to reporting included lack of time and knowledge of reporting processes. To test the magnitude of these factors, further research should be conducted among a larger representative group of healthcare professionals across Australia.

The participants’ recall of training in vaccine safety suggests that there is a need to increase education and training in vaccine and drug adverse events and current reporting methods, at both undergraduate and postgraduate levels, particularly for medical professionals. Specific strategies for updating knowledge should be implemented via the professional relevant accreditation bodies and continuing medical education programs.

The surveillance system and its methods for reporting should be easy to access, widely promoted and “user friendly” to both health professionals and consumers with formats for reporting designed so that the system is accessed effectively in different work settings. The limitation of any passive surveillance system is the submission of reports and the barriers identified in this study should be addressed as the Australian system is strengthened.

## Competing interests

The authors declare that they have no competing interests.

## Authors’ contributions

AP with the supervision of MG, ABM and HM developed the study design, performed the data collection, data analysis and wrote a draft manuscript. ABM assisted in data analysis. The drafts of this article were revised critically by all authors. All authors have contributed to the draft and approved the final version of the manuscript.

## Pre-publication history

The pre-publication history for this paper can be accessed here:

http://www.biomedcentral.com/1472-6963/13/313/prepub
